# Nuclear Mechanotransduction at the Crossroads: How Membrane Receptors Remodel the Perinuclear Cytoskeleton to Drive Cancer and Disease

**DOI:** 10.7150/ijbs.127556

**Published:** 2026-06-25

**Authors:** Michela Sgarzi, Elisa Montacci, Martina Mazzeschi, Alessandra Morselli, Mattia Lauriola

**Affiliations:** 1 IRCCS Azienda Ospedaliero-Universitaria di Bologna, Bologna, Italy.; 2 Department of Medical and Surgical Sciences (DIMEC), University of Bologna, Bologna, Italy.

## Abstract

Growing evidence indicates that nuclear architecture is severely altered in many pathological contexts, primarily in cancer, with major implications for chromatin arrangement and, consequently, gene expression. Actin microfilaments located in the perinuclear region at the apical surface of cells, collectively known as the “perinuclear actin cap”, integrate mechanical and biochemical cues from the cell membrane and translate them into compressive forces acting on the nuclear envelope, thereby modulating nuclear shape and size. In concert with well-established mechanotransduction paradigms, these highly dynamic and finely tuned stress fibers are emerging as key players in several biological processes - from cell migration to sensing of the surrounding microenvironment - with significant implications for development, genetic disorders and tumor progression. However, how classic pathogenetic mechanisms intersect with perinuclear actin remodeling remains mostly unknown. In this review, we will describe in detail the unique functional and structural features of perinuclear actin stress fibers and recapitulate current knowledge on their upstream regulation by membrane receptors signaling. Finally, we will explore how alterations of the perinuclear actin cap may contribute to different pathogenetic processes, with a particular focus on cancer progression and metastasis.

## 1. Introduction

The shape and structure of the cell nucleus are increasingly recognized as critical factors in cell biology, especially in the context of cancer. Changes in nuclear shape are often among the earliest indicators of cellular transformation, and these alterations have profound impacts on cancer progression, metastasis, and even therapeutic response 1. Many biophysical approaches have been used to clarify how the nucleus size and shape are determined in eukaryotic cells. Nowadays, the outdated nucleoskeletal theory 2, which proposed the mass of DNA and the level of its folding as main determinants of the nuclear volume, has been replaced by the model that nuclear dimensions are causally associated with the global cell size 3. Most of the mechanisms that have been proposed to regulate nuclear architecture are linked to nuclear envelope (NE) structural molecules, including the control of availability and organization of lamins or nuclear pore complex (NPC) components 4. Moreover, several processes were found to considerably impact on nuclear shape. First and foremost, the forces exerted by the cytoskeleton on the nuclear surface, which substantially modulate nuclear shape and size. In this context, actin microfilaments have been recognized as the major cytoskeletal components involved in the regulation of nuclear morphology 5, leading to the recent identification of a special subset of stress fibers capable of integrating cues from the cell periphery and physically translating them into nuclear squeezing or expansion: the “perinuclear actin cap” 6-9 or, more generally, “perinuclear actin fibers”. Here, we will review what is known so far about the actin cap molecular structure, as well as the pathophysiological processes involving perinuclear fibers dynamics. Ultimately, we will analyze well-established paradigms and the latest findings concerning upstream modulation of perinuclear actin fibers arrangement in normal and transformed cells, and we will associate them with key cellular processes like mechanotransduction and migration.

## 2. Overview of the perinuclear actin cap

### 2.1. Perinuclear actin cap structure

**Figure 1:**
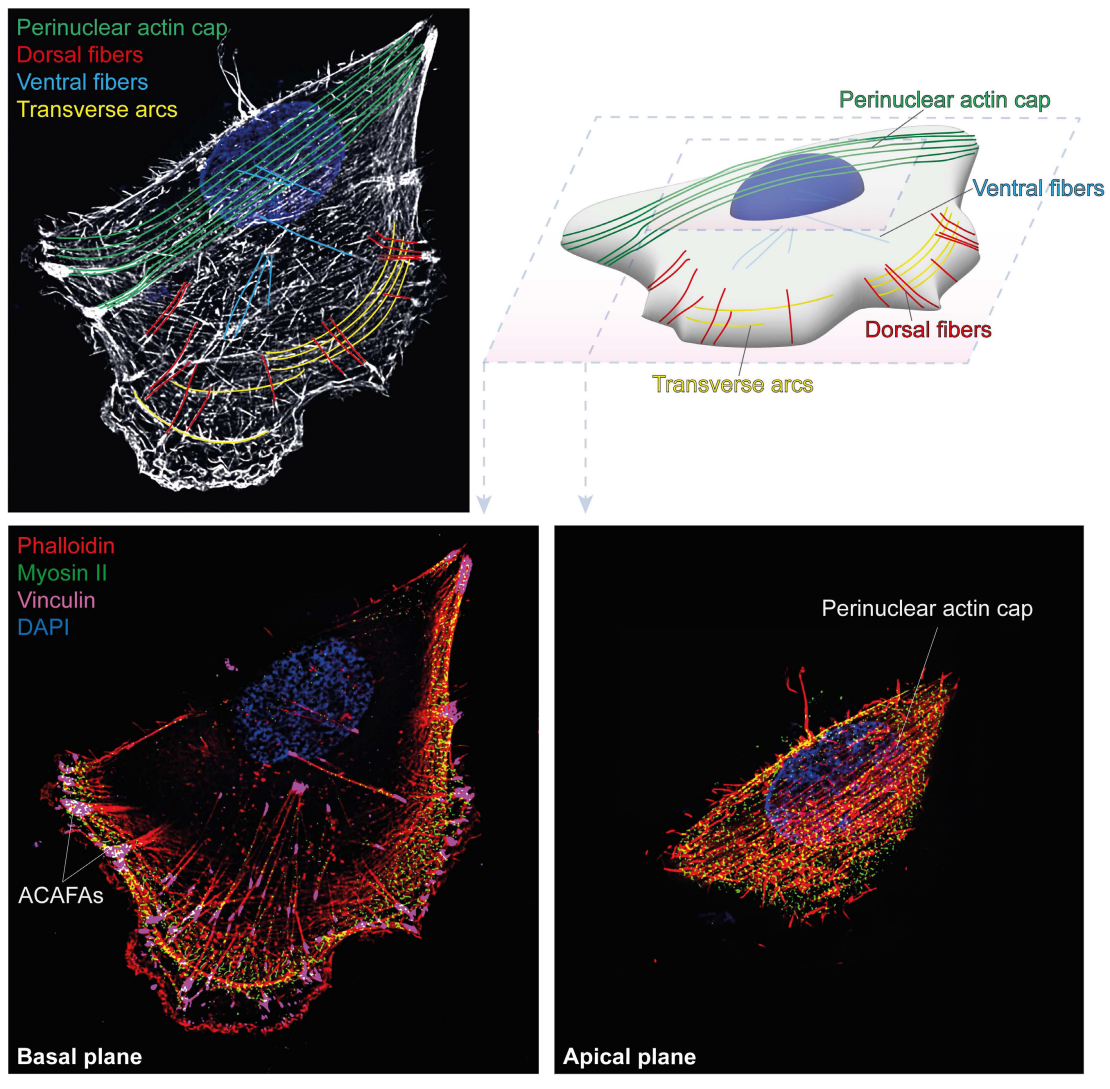
** Spatial organization of actin stress fibers in adherent cells.** Maximum-intensity projection of super resolution images of MCF10A cells stained with phalloidin highlights distinct stress fiber subtypes (color-coded): dorsal stress fibers, transverse arcs, ventral stress fibers and the perinuclear actin cap (**top left**). A schematic 3D representation of the same cell illustrates their spatial arrangement within the cell body (**top right**). Basal (**bottom left**) and apical (**bottom right**) planes show actin together with myosin II and vinculin, highlighting actomyosin contractility and adhesion-associated structures, including actin cap-associated focal adhesions (ACAFAs). Nuclei are counterstained with DAPI.

The cytoskeletal apparatus responsible for the regulation of nuclear shape during interphase was described for the first time in 2009, by Wirtz and collaborators, who identified a set of parallel stress fiber filaments located in the perinuclear region of cells and forming a curved shell above the apical surface of the nuclear envelope 6. The first images of the perinuclear actin cap were obtained in mouse embryonic fibroblasts (MEFs) cultured on adhesive fibronectin-coated micropatterns with various widths. This set of thick apical actin fibers covering the nucleus displays an angle of about 45° from the basal stress fibers. The actin cap was visualized in around 40% of MEFs cultured on unpatterned surfaces, with the remaining 60% of cells showing absent or disrupted caps, as a result of the natural disassembly of actin cap fibers induced by cell mitosis 6. Structurally, the actin cap filaments were quickly found to be linked with non-muscle myosin II (NMIIs), leading to their recognition as part of the actin stress fiber family 6. Indeed, myosin light-chain kinase (MLCK)-mediated phosphorylation of NMIIs in the perinuclear area is required to activate actomyosin contractility and maintain the shape of the actin cap, as treatment with the MCLK inhibitor ML-7 is sufficient to markedly increase apical fibers anisotropy 10. At the cell periphery, the actin cap fibers display terminal end tips decorated by vinculin-rich focal adhesions, renamed as “actin cap associated focal adhesions” (ACAFAs), while at the nuclear envelope, the cap filaments physically contact the nucleus through the Linker of Nucleoskeleton to Cytoskeleton (LINC) 6,9. These unique features led to the recognition of the perinuclear actin cap as a distinct subtype of stress fibers, visibly discernible from the classical stress fiber populations previously described, at first for its localization in the cell, and then for additional functional differences identified in subsequent studies. Ventral stress fibers, the primary contractile structures in interphase cells, are located at the basal plane of the cell; while being anchored to focal adhesions at both ends like the actin cap, completely lack the direct physical connection to the nuclear envelope. On the other hands, dorsal stress fibers, which are again localized on the apical plane, typically extend diagonally to the leading edge of migration toward the nucleus, and importantly lack intrinsic contractility due to the absence of myosin II association. Finally, transverse arcs consist of curved, contractile actin bundles that are not attached to focal adhesions, except indirectly through their interaction with dorsal stress fibers 11-13 (**Figure 1**).

**Figure 2:**
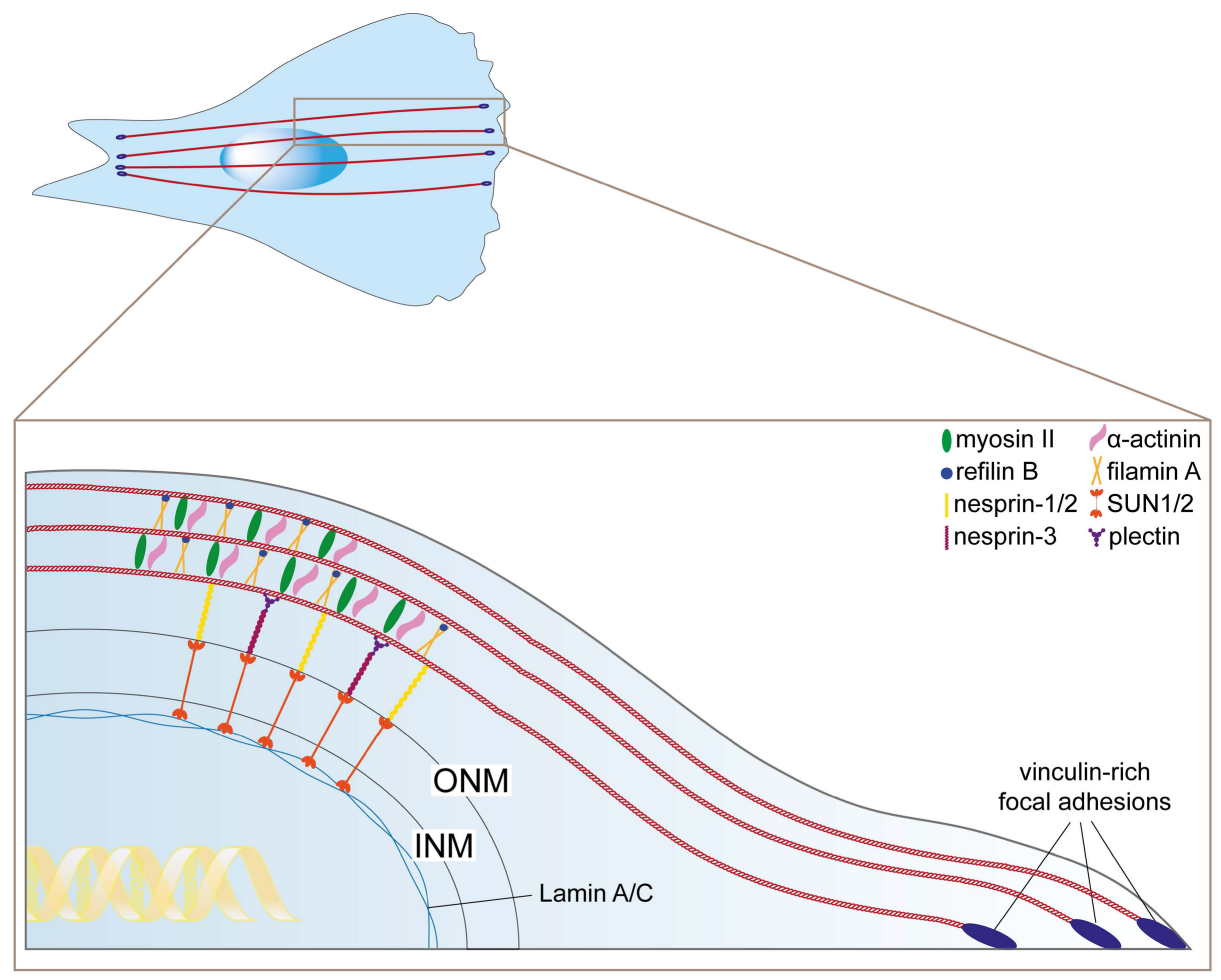
** Actin cap associated proteins.** The perinuclear actin fibers (red) are bundled by filamin A units (light orange) that exert a bundling function in association with refilin B (blue). The actin cross-linking protein a-actinin (pink) is required to stabilize the actin cap, while myosin II (green) confers contractility. The physical link with the nuclear lamina is ensured by the LINC complex components nesprin-1/2 (yellow) and SUN1/2 (dark orange), contacting actin and lamin A/C (light blue), respectively. Nesprin-3 (magenta) contributes to the anchoring of the actin cap to SUN1/2 through its interaction with plectin (purple). At the cell periphery, each perinuclear actin fiber terminates with vinculin-rich focal adhesions named ACAFAs (actin cap associated focal adhesions, dark blue).

Starting from these initial findings, further reports contributed to the definition of the actin cap structural arrangement. Concisely, the sets of proteins that associate with the F-actin filaments to form the final structure of the perinuclear actin cap are summarized in **Table 1** and **Figure 2**. To date, perinuclear actin cap has been studied mainly in fibroblasts. However, actin cap-like structures have also been described in epithelial systems, where they share similar architectural and functional features 14,15. Thus, while structural characterization in epithelial cells remains less extensive, the current literature supports a broader relevance of the actin cap beyond mesenchymal models. Moreover, the classical perinuclear actin cap structure has been well characterized on flat 2D substrates, but cells embedded in 3D matrices adopt non-flattened morphologies and may form different cytoskeletal structures. However, functional analogs exist: actin bundles connected to the nucleus via LINC complexes in 3D serve a similar mechanical role 16. These findings indicate that perinuclear actin organization is a conserved feature in 3D environments, although its structural arrangement in physiological and *in vivo* settings warrants further investigation.

### 2.2. Actin cap associated focal adhesions (ACAFAs)

Focal adhesions (FAs) are multi-protein structures found at the terminal end of stress fibers and responsible for the physical connection with the extracellular milieu, through which actomyosin-based tensions are translated into cell displacement 20. In 2012, Kim and colleagues made a first distinction between the focal adhesions that end the actin cap cables and those that terminate all the other stress fibers 9. Compared to conventional focal adhesions, the actin cap associated focal adhesions (ACAFAs) form around the 30% of all FAs and have a distinct shape, being larger and longer than conventional FAs. ACAFAs are rich in vinculin and activated focal adhesion kinase (FAK), and always show a peripheral localization, at the terminal tips of actin cap fibers 9. Having a crucial role in the anchorage of actin cap fibers, the loss of ACAFAs itself is sufficient to induce a complete perinuclear actin cap disruption 9.

### 2.3. Linker of nucleoskeleton and cytoskeleton (LINC) complex and nuclear lamina

The LINC complex is a multiprotein structure connecting the cytoskeleton to the nuclear lamina, originally described in two parallel reports by Padmakumar 21 and Crisp 22. Over the years, the contributions of many other authors allowed to fully understand the architecture and function of the LINC complex 23-27. The main components of the LINC complex are Sad1 and UNC84 Domain Containing 1 and 2 (SUN1/2) proteins and nuclear envelope spectrin-repeat proteins (nesprins). SUN1/2 N-terminals are directly connected with the nuclear lamina, while C-terminal SUN domains contact the Klarsicht/ANC-1/Syne Homology (KASH) domains of nesprins, on the surface of the perinuclear space. Nesprins extend through the outer nuclear membrane (ONM) and finally interact with the cytoskeleton. While giant nesprins (nesprin-1 and nesprin-2) bind perinuclear actin cap fibers, nesprin-3 is linked to intermediate filaments (IFs) and nesprin-4 to microtubules (MTs) 26. Despite lacking an intrinsic actin-binding domain (ABD), nesprin-3 was found to functionally interact with nesprin-1/2 5 and plectin 28,29 ABDs, thus indirectly contributing to the anchoring of actin cap fibers. A fifth, germ-cell specific nesprin (KASH5), harnesses the dynein-dynactin complex to support chromosomes displacement during meiosis 30. The integrity of the whole LINC complex system, including a proper arrangement of the nuclear lamina, is crucial to the formation of the actin cap. Indeed, a complete loss of actin cap organization was induced by displacing nesprin-2 from the nuclear envelope through EGFP-KASH2 introduction 6. Impairment of the LINC complex by siRNA-mediated knockdown of SUN1/2 or SYNE1/2, encoding for giant nesprins-1 and 2, has been successfully employed as experimental tool to specifically induce actin cap abrogation without using actin-affecting drugs 15,31. In addition, nesprin-3 knockdown was found to prevent actin cap formation upon shear stimulation, confirming the indirect interaction with perinuclear actin fibers 8. Finally, a proper arrangement of the nuclear lamina is crucial to the formation of the perinuclear actin cap, as suggested by the complete loss of actin cap - but not basal stress fibers - alignment in cells harboring alterations of A-type lamins 6. Moreover, lamin A/C proper organization mediates the role of the actin cap as shield against extracellular mechanical stresses, as will be further discussed later 17.

## 3. Perinuclear actin cap functions

### 3.1. Actin cap role in nuclear shaping

The contribution of the actin cap to the regulation of nuclear shape was first suggested through experiments using low doses of Latrunculin B (LatB). This treatment selectively disrupted the rapid polymerization of actin cap filaments, while leaving the long-lasting basal stress fibers unaffected 6. In MEFs, the selective abrogation of the actin cap led to an increase in nuclear thickness, suggesting that the actin cap fibers could flatten the nucleus towards the basal plane by exerting vertical constraints on the nuclear envelope. Likewise, nuclear bulging in actin cap-bearing cells was obtained by treatment with inhibitors of the actomyosin contractility 6. Similar observations were reported in endothelial cells, where the actin cap exerts vertical compressing forces on the nucleus, promoting its basal flattening, and contributing to the regulation of nuclear geometry and mechanical polarization 32. The actin cap was also found to impact on the inner structure of interphase nuclei. Indeed, the viscous lamin A/C molecules relocate on the apical section of the nuclear envelope to absorb the pressure imposed by the actin cap fibers. This pressure is intrinsically higher than the one generated by basal stress fibers due to more active myosin II 9 and the physical connections with both the nuclear compartment and the cell periphery 33. Interestingly, hyper-acetylated histones, commonly associated with transcriptionally active chromatin, were found to apically polarize in the nucleus upon actin cap formation 33. Mechanistically, this effect likely results from an unbalanced accessibility/distribution of histone modifying enzymes (e.g. histone deacetylases), as their nucleocytoplasmic shuttling was shown to be largely sensitive to modifications in nuclear matrix architecture or actomyosin contractility itself 34,35. Likewise, perinuclear actin cap fibers were found to be physically associated with heterochromatin foci, suggesting a functional role of the actin cap in dynamically regulating chromatin remodeling through a direct force transduction process 36. Notably, the differential role of apical actin compared to basal fibers and microtubules is very well conserved evolutionary, suggesting a shared mechanism of nuclear structural regulation mediated by the perinuclear actin network. Indeed, in *Drosophila melanogaster* cells, the nuclear morphology is maintained by a fine balance between microtubule-derived forces, which induce nuclear vertical expansion, and perinuclear apical actin tensions prompting nuclear flattening 37. Perinuclear actin cap perturbation was achieved by knocking down the nesprin ortholog gene Msp300, producing the same effects observed in human cells: increased nuclear height and core histone mobility as well as upregulation of cell motility gene groups 37, as will be discussed later.

Finally, by modulating nuclear compression, the actin cap indirectly regulates nuclear and cell stiffness. For instance, in rat mesenchymal stem cells (MSCs), the actin cap was found to sustain the surface tension, while the pharmacological inhibition of the actin cap was sufficient to decrease surface stiffness and increase the nuclear thickness, as measured by atomic force microscopy (AFM) 38. While the actin cap plays a role in the mechanical regulation of nuclear shape, the nucleus is additionally exposed to forces arising from the complex cytoskeletal network, where MTs and IFs cooperate with perinuclear actin. MTs exert mainly compressive and tensile forces on the nucleus, thus influencing nuclear invaginations and morphology via motor proteins (e.g. dyneins) and the LINC complex 39,40 and synergize with actomyosin to regulate nuclear mechanics 41. Moreover, in mechanically stressed cells such as adult cardiomyocytes, MTs form a perinuclear cage that transmits forces to the nucleus representing a dominant source of NE damage when nuclear lamina integrity is compromised, independently of actomyosin-LINC linkage 42. IFs, especially those composed of vimentin, also form a perinuclear filamentous network that mechanically resists deformation, regulates nuclear shape and contributes to nuclear stiffness, limiting excessive nuclear damage particularly during migration in confined environments 43,44. Of note, abnormal perinuclear accumulation of vimentin in cancer contexts was found to contribute to nuclear dysmorphia and genomic instability 45. Moreover, key crosslinking proteins (e.g. plectin) are able to integrate contributions from different cytoskeletal components to modulate nuclear morphology 46,47. Overall, recent literature converges on a model in which nuclear shaping arises from an interconnected network in which actin, MTs and IFs, contribute to mechanical coupling between the cytoskeleton and nucleus. Future work should address how the relative contributions of distinct cytoskeletal structures to nuclear mechanics are hierarchically organized across different cell types, mechanical contexts and temporal scales.

### 3.2. Actin cap role in nuclear movements and cell motility

The actin cap dynamic assembly determines directionality and polarization during cell migration. For example, the typical stop-and-go motion that characterizes mesenchymal migration is finely tuned by actin cap formation and dismantling 7. Indeed, the cap fibers were shown to harness the nucleus during fast directional movements of cells, by forcing the cell to move toward the direction of perinuclear actin filaments for 30'-2h by preventing nuclear rotation. This mechanism links the actin cap to directional migration. Most likely, in order to favor cell migration toward a certain direction, the actin cap stabilizes the main lamellipodium thanks to the association with bigger and longer-lasting focal adhesions 7. Interestingly, dismantling the actin cap is required when cells slow down to change their orientation and repolarize the nucleus to follow the new direction. Nuclear reorientation was suggested to rely upon a dynein light intermediate chain 2 (LIC2), consequent to the actin cap abrogation 7. Maninova et al. expanded this model by showing that the actin cap cables can derive from dorsal fibers, peripheral stress fibers and transverse arcs, which form a grid that continuously flows towards the apical cell center. In the perinuclear area, the actin fibers attach to the nuclear envelope through the LINC complex to form a mature actin cap, contributing to the reorientation of the nucleus in the same direction as the perinuclear filaments 48. Interestingly, the role of the actin cap in positioning and reorienting the nucleus appeared conserved also in other organisms. In *Drosophila melanogaster*, a filamin-containing perinuclear actin meshwork induces nuclear turning during the process of dumping, when nurse cells eject their content into oocytes by contraction 49.

As cancer cells acquire the ability to invade and spread, changes in nuclear shape are essential to facilitate migration. Cells with deformable, plastic nuclei can squeeze through dense tissue environments, supporting their dissemination to distant sites. This nuclear plasticity enables cancer cells to adapt to various mechanical stresses encountered in the extracellular matrix and blood vessels. Therefore, an aberrant nuclear shape is not just a byproduct of cancer but a functional feature that aids in metastasis. When in complex and heterogeneous microenvironments, cancer cells are able to shift from a mesenchymal migration, aligned with the axis of actin cap fibers, to amoeboid-like motility, which is recognized as the preferred migratory strategy for cells under confinement 50,51. Upon the switch, the highly tensed perinuclear actin cap filaments and the long-lasting ACAFAs characteristic of elongated mesenchymal cells may disappear to leave room for short-term adhesions to the ECM, blebbing and an overall rounded phenotype typical of amoeboid cells. Under amoeboid motility, an actin cap-independent mechanism involving Arp2/3-driven actin polymerization in the perinuclear area was reported to foster cell deformability during confined migration 52. Moreover, myosin II, LINC complex and FAs were found to be completely absent in this process, supporting the idea that perinuclear actin cap alignment could be useless or counterproductive in ameboid migration 52. In metastatic cancer cells, the disassembly of the actin cap could be a driver mechanism responsible for the switch from mesenchymal-to-amoeboid transition (MAT) depending on the surrounding environment. Actin cap disruption may increase nuclear deformability of metastatic cancer cells during extra- and intra-vasation, fostering cell squeezing 53. Nevertheless, the field remains controversial, as additional reports suggest that in certain cell types the actin cap presence may contribute to confined migration. For example, actin cap assembly driven by Palmdelphin (PALMD) activation has been reported to facilitate the confined motility of HUVECs through microchannels 54. However, strategies of nuclear positioning can widely differ between cell types, with hematopoietic cells often displaying a leading nucleus and fibroblasts or epithelial cells generally positioning the nucleus at the rear 55-57. Likewise, in complex microenvironments cells can adopt mechanistically distinct modes of 3D migration depending on their physical properties and their adhesive and contractile state 58,59, suggesting that the role of the actin cap in 3D may similarly depend on the cell type.

**Figure 3:**
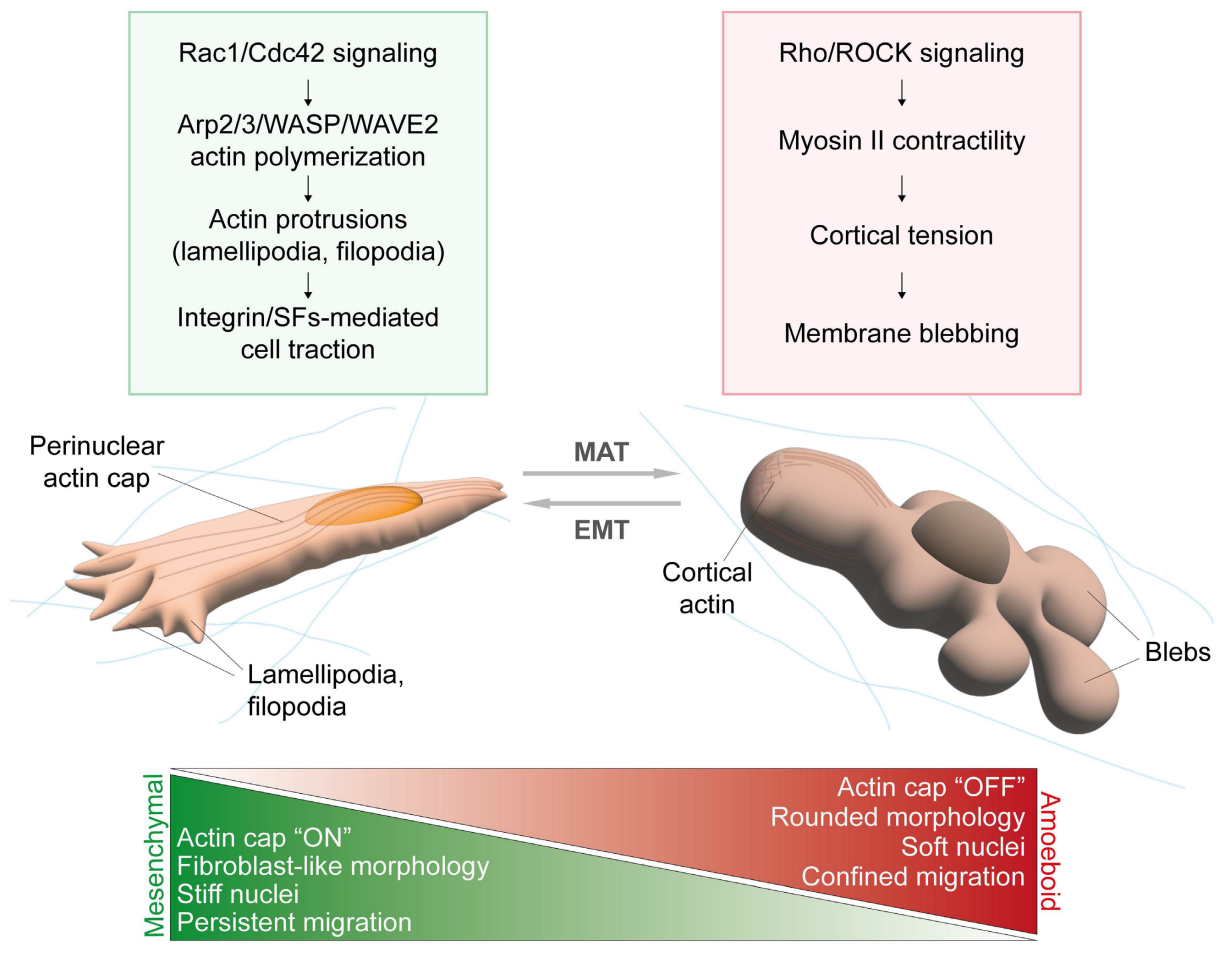
** Actin cap and mesenchymal versus amoeboid phenotype.** In cells moving through mesenchymal-like motility, GTPases Rac1 and Cdc42 primarily guide the formation of actin protrusions (lamellipodia, filopodia) at the leading edge via Arp2/3 and WASP/WAVE proteins, although additional mechanisms may contribute depending on the 3D context 61. Integrin-based adhesion to the ECM stabilize lamellipodia and fliopodia at the leading edge and connection with contractile stress fibers (SFs) transmit traction forces allowing cell movement 62,63. In this process, the actin cap is responsible for guiding persistent and directed cell displacements and nuclear translocation 7. Actin cap-bearing cells usually display an elongated and fibroblast-like cell morphology, together with flat and stiff nuclei. Cells shifting towards an amoeboid-like phenotype perform fast and undirected cell movements, particularly suitable for confined migration in complex environments. Here, cell motility is mainly guided by RhoA/ROCK-induced activation of Myosin II, which enhances cortical actomyosin contractility generating tension in the cortical region. High intracellular pressure may induce membrane detachment from the cortex and formation of blebs, which drive cell translocation 62,63. In the case of amoeboid cells, the actin cap must be disrupted to allow the acquisition of a rounded cell morphology, associated with nuclear expansion and softening.

Moreover, during confined migration, the actin cap has been considered both as a protection against mechanical tensions acting on the nucleus 54 and as a primary source of nuclear envelope stress due to the intensification of intranuclear pressure derived from perinuclear actin-dependent nuclear confinement, causing chromatin herniation and nuclear membrane instability 60 - although a possible technical limitation to the last phenotype was the employment of U2OS cells that are cancer cells lacking the actin cap 9. To sum up, whether the actin cap positively or negatively influences confined migration remains an open question; however, its role might be closely tied with the mesenchymal-to-amoeboid transition (**Figure 3**).

### 3.3. Actin cap and mechanotransduction

*Mechanosensing*, the ability of cells to detect physical cues from their microenvironment, and *mechanotransduction*, the process through which these stimuli are translated into intracellular biochemical signals, are emerging as key determinants of cell behavior in physiological and pathological contexts 64,65. In this perspective, the actin cap occupies a strategical position, as it represents the only subset of actin stress fibers linking the nuclear compartment to the surrounding cell surface. Unlike conventional FAs, ACAFAs are known to participate to a fine-tuned mechanosensing process that allows adherent cells to perceive exceptionally tiny changes in the topography of the extracellular environment. Indeed, the sensitivity to changes in substrate compliance was found to be different between conventional FAs and ACAFAs. While all FAs are dismantled in the presence of hyper-soft substrates (kPa < 1), ACAFAs are the first one to disappear already in milder substrate stiffness, suggesting a role in early mechanosensing 9. Other adhesion structures like fibrillar adhesions (FBs) also exhibit pronounced mechanosensitivity and respond rapidly to subtle changes of the substrate stiffness. Unlike ACAFAs, FBs are centrally located and arise from maturation of FAs, evolving into α5β1 integrin/tensin-rich structures tightly associated with fibronectin in the ECM 66,67. Their presence is independent of actomyosin-mediated tension 67. Notably, FBs exhibit a mature elongated phenotype already at a very low substrate stiffness, and further elongate as stiffness increases 68. Recent work showed that FBs can sustain nuclear morphology and mechanosensing in fibroblasts when actin cap organization and actomyosin contractility are disrupted 69. Altogether, these findings suggest that FBs may represent a complementary mechanism shaping the early mechanotransduction landscape.

The early response to mechanical cues mediated by the actin cap is not limited to substrate softening. Indeed, the actin cap apparatus allows to sense flow-induced shear stresses about 50 times better than basal stress fibers 8. Moreover, upon substrate stretching, the actin cap activates an “emergency” mechanism protecting nuclear integrity, dependent on a correct organization of lamin A/C 17. Indeed, according to computational and biological evaluations, uniaxial cyclic strain can stimulate the thickening of the cap filaments, that co-localizes with indentation marks on nuclear lamina following each perinuclear fiber. Lamin A/C mutations completely abrogate the actin cap-mediated protection over stretch-induced nuclear deformation, indicating a major role of the nuclear lamina in facilitating the actin cap dissipation of extracellular tensions. In this context, treatment with myosin light-chain kinase inhibiting drugs (ML-7) or nesprin-2G knockdown impairs actin cap effectiveness as well 17. To sum up, mechanical forces applied to the cell promote the thickening of the actin cap to evade nuclear deformation, but only in the presence of (I) an unaltered nuclear lamina, (II) a properly formed LINC complex and (III) functional myosin II units to guarantee filaments contractility.

In line with these data, Tamiello et al. unveiled a differential response of basal and perinuclear actin in vascular derived cells (HVSCs) exposed to topographical and mechanical cues at the same time, as often happens *in vivo* 70. To this end, they analyzed the orientation of stress fibers in cells cultured in micropost arrays, which induce alignment of stress fibers along the major axis, phenomenon known as contact guidance. Under these conditions, they applied uniaxial cyclic straining for 19h and observed that specifically the actin cap fibers, rather than basal stress fibers, reorient away from the strain direction, a phenomenon known as “strain avoidance”. Apparently, the actin cap fibers are more prone to strain avoidance while basal stress fibers preferentially follow contact guidance cues 70. As a result, cells orient their apical fibers perpendicular to the direction of the stretch, likely to minimize deformation.

LMNA-deficient fibroblasts that cannot organize the actin cap retain the capability to respond to topographical cues only, losing the strain avoidance response when subjected to uniaxial strains. These results suggest that the actin cap contributes more critically to the response of a cell to the mechanical stimuli, than to its ability to perceive the surrounding environment through mechanosensing 71. Similar results were obtained in lung epithelial cells, where cyclic stretches were found to strengthen the formation of perinuclear and peripheral stress fibers while reducing dorsal stress fibers and transverse arcs number 72.

The actin cap was also found to be involved in driving cell response to mechanical stimuli. In details, cell traction forces in fibroblasts appeared increased over the perinuclear area compared to the periphery of the cell, identifying the actin cap as the main mediator of nuclear displacement, in an alpha-actinin/zyxin-dependent manner. The high perinuclear forces produced by the actin cap were found to correlate with increased YAP nuclear import, which was indeed dependent on the perinuclear contractility 73. Similarly, the knock-down of actin cap alignment mediators such as STEF/TIAM2 impairs YAP/TAZ-mediated mechanotransduction 74. Indeed, YAP nuclear localization can be driven by increased actin cytoskeletal tensions, which were shown to antagonize Hippo pathway-mediated YAP phosphorylation and cytosolic retention 75,76. Notably, perinuclear stress fibers alignment can prompt YAP nuclear translocation also independently of the Hippo pathway. A possible mechanism may be associated with the intensification of the stiffness of perinuclear actin fibers, that could push on the nucleus and physically increase the opening of nuclear pores, thus boosting YAP nuclear translocation 77. However, protracted nuclear stiffening induced by significant actin cap intensification upon prolonged shear stress gradually diminishes nuclear deformability leading to YAP export from the nucleus at later time points 78. Together, these findings point to a biphasic response, in which early mechanical cues favor YAP entry to the nucleus, whereas sustained actin cap-mediated stiffening eventually elicits the opposite effect.

**Figure 4:**
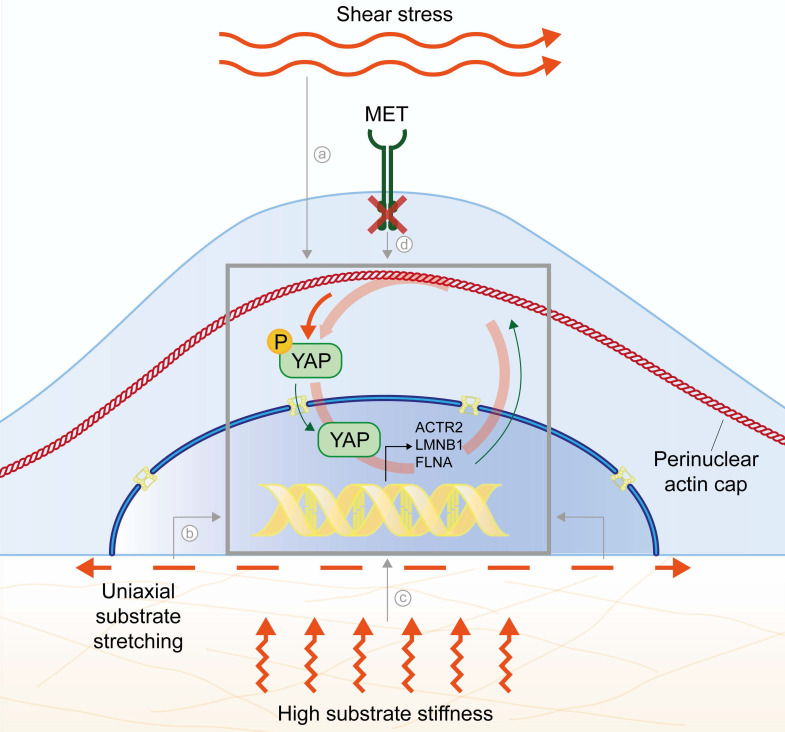
** Mechanoregulatory feedback loop between the actin cap and YAP.** Several mechanical cues prompt perinuclear actin cap alignment and intensification: (a) flow-induced shear stress, even at low intensity 8; (b) uniaxial substrate stretching 17,70,72; (c) rigid substrates 9,29; (d) MET inhibition 15. Perinuclear actin cap alignment provides compressive forces prompting YAP nuclear translocation 75,77. Among the target genes of nuclear YAP co-transcriptional activity, actin cap related structural genes (ACTR2, LMNB1, FLNA) further improve perinuclear actin fibers alignment and stabilization 15,31,79.

Despite the time/context-dependent effect of actin cap alignment on YAP nuclear translocation and resulting nuclear stiffening, all these reports support the model of the actin cap as a regulator of YAP-mediated mechanotransduction, especially for what concerns cells capability to discriminate between growth on stiff or soft substrates. Nonetheless, it was recently reported that YAP co-transcriptional activation itself could target genes involved in the organization of perinuclear actin, suggesting a driver role for YAP in the formation of the actin cap 31. Indeed, experimentally-induced YAP depletion in young fibroblasts or physiological impairment of YAP pathway due to ageing in explanted old fibroblasts was found to inhibit actin cap organization and increase nuclear dysmorphia, while YAP activity induction restored the actin cap in old cells 31. Mechanistically, functional screen returned ACTR2, encoding for ARP2 protein involved in actin polymerization, as direct YAP target gene 31. In line with these data, we also unveiled a pathway driven by the hepatocyte growth factor (HGF) receptor, MET, upstream to the actin cap disruption in cancer cells 15. In this model, MET-mediated aberrations of the actin cap were found to correlate with YAP inactivation through cytosolic retention. Notably, the forced introduction of a constitutively active form of YAP harboring 5 mutations on serine residues (YAP5SA) was sufficient to fully restore a functional actin cap, despite the highly aggressive features of MET aberrant cancer cells 15. In line, a comprehensive analysis in liver cells explanted from transgenic mice showed that constitutive activation of YAP is able to foster the production of filamin A protein (FLNA), largely involved in the structural organization of the actin cap 79. Overall, these findings support a causative role for YAP co-transcriptional activity in the expression of genes that drive the assembly of the cap. This, in turn, may enhance YAP nuclear import through compressive forces generated by perinuclear actin reinforcement as previously discussed. The result is a positive feedback loop between actin cap formation and YAP nuclear translocation (**Figure 4**). However, this model should be interpreted with caution since YAP activity can be either repressed or enhanced depending on the specific cancer type 80-82. Indeed, in cancer cells the perinuclear actin cap is frequently disrupted or absent, suggesting that YAP activity alone may not be sufficient to drive cap assembly and that the interplay between actin cap organization and YAP signaling may be tissue- or context-specific.

Notably, the interplay between YAP and the actin cytoskeleton may not be limited to perinuclear actin cap, with very recent works suggesting that other nuclear-associated stress fibers located in the ventral side of adherent cells can impose tensional forces on the nuclear lamina from the basal plane 83. Of note, mechanotransductive feedback loops similar to those observed for the actin cap, where cytoskeletal regulators are themselves transcriptional targets of YAP, have been described, pointing to an integrated crosstalk involving also MTs along with actomyosin network 83,84.

## 4. Upstream to the actin cap

While the architectural organization of the actin cap has been extensively studied 6-8,19, the upstream signals regulating its formation and structure remain unclear. In other words, although we understand how the actin cap is formed and maintained, the specific processes and molecules that influence or drive its organization are not yet fully understood. This is particularly relevant for membrane receptor tyrosine kinases (RTKs), which are well known to play a role in cytoskeleton regulation and cell migration 85-87, yet their specific effect on the actin cap remains elusive.

In mouse epithelial cells, TGF-β-mediated EMT fosters RefilinB/FLNA complexes formation to finalize perinuclear actin cap assembly 19. In line, in mouse fibroblasts, upon RefilinB interaction with Filamin A, the latter switches from an F-actin branching into an F-actin bundling activity, stabilizing perinuclear actin fibers into parallel filaments wrapping around the nucleus to form the actin cap 19.

So far, only few reports proposed upstream regulatory mechanisms acting directly either on actin cap stabilization or on the control of the actin cap assembly. Among other membrane receptors, integrins, the focal adhesions core proteins, are plausible candidates for upstream regulation of actin cap fibers, as integrin-mediated signaling is well established to regulate actomyosin stress fibers assembly through canonical FAK/Src-dependent activation of RhoA/ROCK (refer to reviews on the topic 88,89). Moreover, the role of integrin/zyxin-rich perinuclear adhesions in transmitting forces to the actin cap via the LINC complex has been robustly characterized 73. Within this framework, it is reasonable to hypothesize that signaling axes converging on integrins or other cell surface adhesion receptors, such as CD44, may also participate in regulating actin cap tension or even its formation and stabilization. However, how the canonical integrin-FAK/Src-RhoA/ROCK pathway specifically guides perinuclear actin cap assembly remains unclear and the full signaling axis has yet to be dissected. In this context, Formin HOmology Domain 2-containing (FHOD) proteins, may play a key role. Recent cell biology and structural studies highlighted FHOD1 and FHOD3 as potent actin bundlers that associate with nesprin spectrin repeats of the LINC complex, thereby providing an additional actin binding site adjacent to nesprins that may enhance actin-LINC engagement. Through this interaction, FHODs mechanically couples perinuclear actin arrays to the nuclear envelope and have been shown to contribute to nuclear positioning in fibroblasts and cardiomyocytes 90-93. Of note, FHODs activity is regulated by phosphorylation via ROCK and Src kinases 90,94,95, consistent with upstream adhesion-dependent signaling, and by ERK1/2 93, suggesting potential links to other receptor-mediated cascades. While these data support a mechanistic role for FHODs in reinforcing cytoskeletal-nuclear physical connection, clear evidence that FHODs assemble the contractile actin cap downstream of integrin or other membrane receptor signaling is still lacking. Indeed, most studies, such as Antoku et al., examined FHOD1 bundling activity on TAN lines and dorsal stress fibers, rather than on the actin cap 93. For an in-depth discussion of differences and similarities between actin cap and TAN lines, we refer readers to 96. As previously mentioned, also YAP, probably the most studied mediator of mechanosensing and mechanotransduction, was found to prompt actin cap alignment 15,31, which might explain its correlation with persistent cell motility, a typical feature of actin cap-bearing cells 97. Moreover, recent findings showed that YAP downregulation promotes fast amoeboid migration and high metastatic potential in lung cancer cells, suggesting that the actin cap loss triggered by YAP inhibition might be essential to drive mesenchymal-to-amoeboid transition 98. Recently, we proposed the involvement of the receptor tyrosine kinase MET in the dismantling of the actin cap through YAP repression.

MET is involved in cytoskeleton remodeling via Rac1 signaling, both by recruiting Rac1 to the plasma membrane 99 and by endosome signaling in the perinuclear area 100. However, the outcome depends on the duration and intensity of the signal. While acute ligand-mediated MET activation triggers classical mesenchymal-like cell morphology and migration, sustained receptor hyperactivation driven by gene overexpression promotes cell rounding 101 and amoeboid shape. This suggests that the effects of MET on the actin cytoskeleton differ depending on the mode of activation. In our work, we described a deep perinuclear actin rearrangement upon MET hyperactivation, not only in gastrointestinal cancer cells naturally expressing constitutive aberrant forms of MET (LoVo, GTL16), but also in normal epithelial cells (MCF10A) engineered to carry the translocated hyperactive TPR-MET protein 15. In our hands, the knock-out of MET was sufficient to restore a functional actin cap despite the cancer background of the cells, while the rescue of the receptor reproduced the original aberrant morphology. Following the actin cap alterations, nuclear shape and cell migratory behavior were affected, with cells characterized by considerable nuclear height and meandering random migration 15. We also proved the involvement of YAP in the actin cap organization, with a complete inhibition of its activity and cytosolic translocation upon MET hyperactivation 15, leading to the loss of the perinuclear actin cap and amoeboid transition.

Our work linked for the very first time this membrane receptor to the selective control over actin cap fibers, suggesting that signaling-mediated pathways could contribute to the actin cap regulation in concert with extra- and intracellular physical cues. However, the extent to which this regulation is causally dominant across cancer contexts requires further elucidation, particularly *in vivo* and in patient-derived settings.

**Figure 5:**
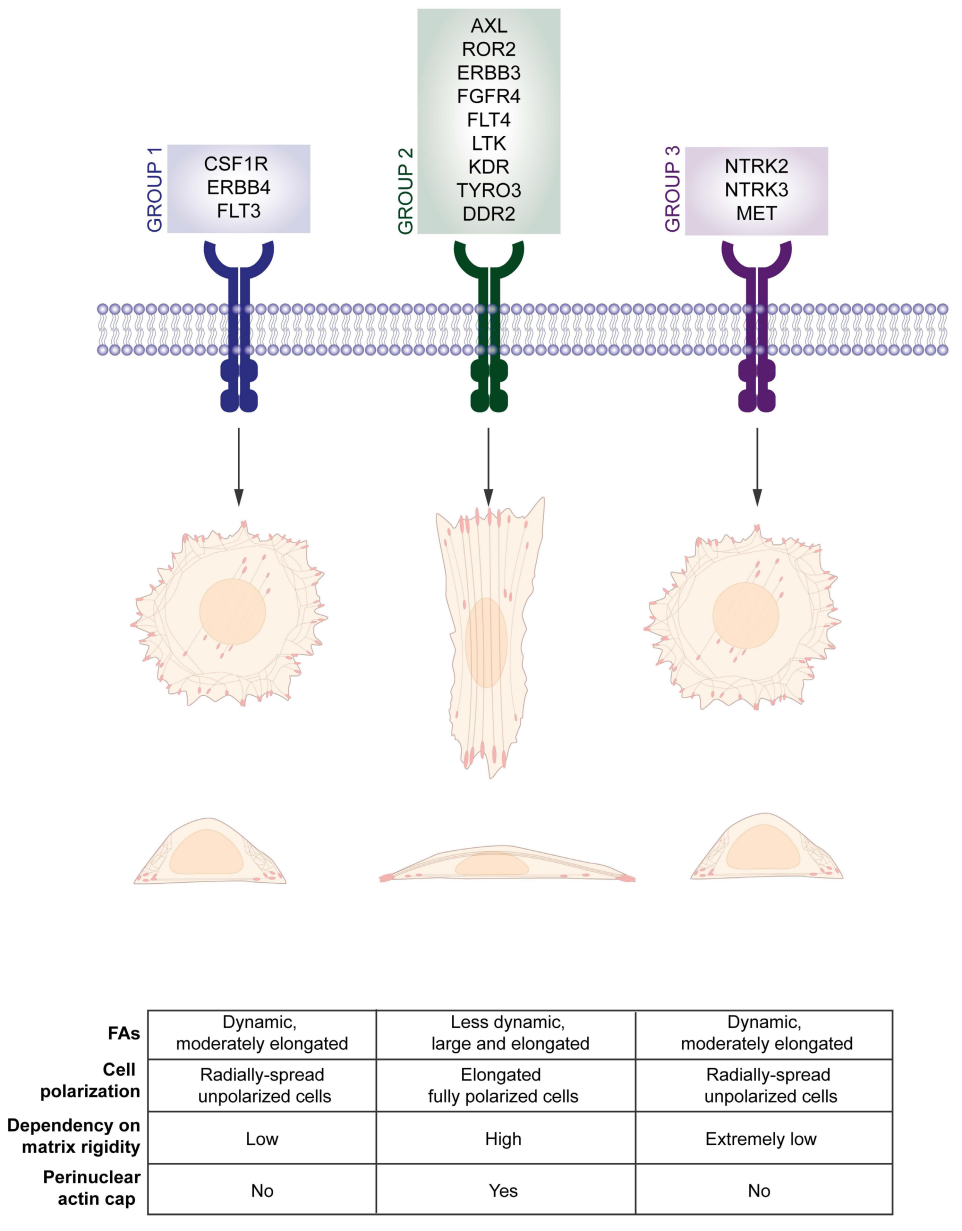
** Receptor tyrosine kinases as upstream mediators of cell polarization and actin cap alignment.** Upregulation of group 1 and group 3 RTKs, involved in early stage of focal adhesions development and loss of matrix rigidity dependency, respectively, reduces cell polarization and likely induces actin cap disruption. On the contrary, upregulation of group 2 RTKs prompts cell elongation and polarization on rigid substrates, possibly through the induction of perinuclear actin cap alignment 106.

Notably, other RTKs are known to influence YAP activity through pathways that intersect with cytoskeletal dynamics, often with opposite effects compared to what is observed for MET receptor. For instance, EGFR has been shown to enhance YAP activation by interfering with the activity of inhibitory kinases of the Hippo pathway 102-104. Since RTK signaling can both modify actin cytoskeletal architecture and converge on mechanosensitive hubs like FAs and Rho GTPases 105, it is plausible that RTK-induced YAP activation may feedback to influence actin cap assembly or stability. However, direct evidence for such a loop remains to be established, and studies performed so far suggest that different RTKs could play differential roles in modulating actin cap-YAP interplay. Indeed, a knock-down screening performed on more than 80 different protein tyrosine kinases revealed a differential involvement in the regulation of cell polarization, focal adhesions arrangement and stress fibers-mediated mechanosensing of matrix rigidity, phenotypes that can all be ascribed to the proper assembly of the actin cap 106. siRNA screening unveiled that CSF1R, CSK, FER, ERBB4, FLT3 are likely to be involved in the very initial stage of focal adhesion formation; AXL, ROR2, JAK1, ERBB3, PTK9, FGFR4, FTL4, LTK, KDR, TYRO3, DDR regulate cell dependency on matrix rigidity, as focal adhesions number on soft substrates markedly increased when they were silenced; finally MET, NTRK2/3 and PTK2B, hold an opposite role, allowing cells to grow and elongate also on compliant substrates by losing the rigidity-dependent feature 106. Although the authors did not analyze the status of the actin cap fibers in the three subgroups, the knockdown phenotypes suggest that some RTKs likely promote actin cap assembly, whereas MET and NTRK2/3 appear to play an inhibitory role **(Figure 5)**. Likewise, mutations on the gene encoding for the surface receptor MERTK were found to impact on cell shape and directional migration in macrophages, most likely through the disruption of the actomyosin apparatus 107.

Despite these advances, how these different upstream mediators and signaling pathways might cooperate to achieve the final organization and dynamics of the actin cap is still an open question.

## 5. Perinuclear actin cap aberrations as a new pathological mechanism?

The perinuclear actin cap has been described in healthy cells, including fibroblasts (MEFs, HFFs), myoblasts (C212C), endothelial (HUVECs) 6-9,17,29 and epithelial cells (MCF10A) 15. On the other hand, an aberrant perinuclear actin cap as distinctive feature of diseased cells has been reported in an increasing number of pathologic conditions, some of which has been already discussed in the previous paragraphs.

**Table 2** lists the diseases in which alterations of the actin cap have been observed so far.

Laminopathies represent an interesting model for the study of the actin cap modulation and have been widely used to study the actin cap defects and their biological consequences. Indeed, fibroblasts derived from Emery-Dreifuss muscular dystrophy mice models which lack *LMNA* gene lose the anchorage of perinuclear actin fibers to the nuclear lamina, while the basal stress fibers remain unaffected 6,7,17,29. Lack of *LMNA* gene is sufficient to disassemble the actin cap. Notably, Hutchinson-Gilford Progeria Syndrome (HPGS) mutation *Lmna*^l530p/l530p^ induces an even more severe actin cap disruption 6. Interestingly, the lower cancer susceptibility in these genetic disorders suggest that an aberrant actin cap is not causally linked to cell transformation and onset of cancer. Of note, overexpression of the LINC complex protein nesprin-2 in progeria models with LMNA^S143F^ mutations was able to rescue nuclear morphology alterations, suggesting that the loss of NE-actin cytoskeleton connection might contribute to laminopathic phenotypes instead of being a secondary effect of nuclear lamina defects 108.

Interestingly, a defective alignment of the perinuclear fibers was detected also in other rare genetic disorders not belonging to the spectrum of laminopathies, such as Huntington disease (HD). HD affects primarily brain tissue but displays multisystem effects and is caused by mutations in huntingtin gene (*HTT*), without evident connections with cytoskeletal deficiency. Nonetheless, in primary skin fibroblasts explanted from HD patients, the actin cap was found to be robustly disrupted in association with nuclear morphological alterations 109 with many phenotypical similarities with progeria laminopathic models 6. In Spondylocarpotarsal synostosis syndrome (SCT), a case report identified a novel mutation in Refilin A (*RFLNA*) gene in a patient showing typical SCT symptoms but with normal mRNA expression of filamin B (*FLNB*), usually altered in SCT patients as major pathogenetic driver. Given the key role of refilins and filamins in the formation of actin caps, the authors propose the actin cap disruption as a new phenotype that is worth investigating 110.

More generally, together with the main disease-causing mutations, alterations of LINC complex genes were detected in several laminopathies and genetic diseases characterized by nuclear architecture defects, including Duchenne Muscular Dystrophy (DMD), Emery-Dreifuss muscular dystrophy (EDMD) 111,112, recessive spinocerebellar ataxia type 8 (SCAR8) 113, autosomal recessive arthrogryposis 114 and many others. Given the importance of LINC complex integrity in the alignment of the actin cap, it would be worth studying to what extent the perinuclear actin cap is compromised in these conditions and understand whether and how actin cap defects contribute to the diseased phenotype.

Also, accelerated ageing has been correlated to actin cap aberrations in stromal cells, in absence of specific acquired mutations in structural genes related to actin cap or nuclear lamina organization. Indeed, given the previously described role of YAP/TAZ pathway in sustaining actin cap formation, its inactivation following natural decline was shown to be responsible for the alterations in NE architecture and loss of nuclear integrity usually observed in aged fibroblasts 31.

Moreover, developmental defects could arise from actin cap alterations in various stem cell compartments. Interestingly, the actin cap is retrieved only upon cell differentiation. Of note, the presence of Lamin A/C and LINC complex in hESC is not sufficient for the complete formation of the actin cap and the optimal nuclear shaping, which only occurs upon differentiation 115. In line, stem cell reprogramming into induced pluripotent stem cell (IPSC) leads to loss of actin cap organization. Notably, only the perinuclear actin cap showed significant modifications during the differentiation process, not other stress fibers subtypes 115. In mesenchymal stem cells (MSCs), featuring a properly formed actin cap, experimentally-induced loss of the actin cap coordinated nuclear height increase and decreased surface stiffness, but also boosted cell proliferation rate 38. As such, the proper assembly of the actin cap, may represent a true differentiation marker for epithelial and mesenchymal derived cells.

Intriguingly, in mature muscle cells organized into multinucleated syncytia, the actin cap holds an essential role in nuclear positioning and regulation of nuclear shape. This was evaluated in C2C12 myoblasts, which display a cytosolic pool of HP1-gamma associated with actin cap. HP1-gamma knockdown compromised myoblasts differentiation resulting in thin myotubes. Given the nuclear-shaping role of the actin cap, the authors suggest a potential involvement of actin cap disruption in the altered differentiation of myotubes induced by HP1-gamma RNAi 116. Despite the correlative nature of this study, it is noteworthy that the HP1-gamma, which is involved in the differentiation process of muscle cells, is also physically associated with the actin cap.

The role of the actin cap in sensing and responding to mechanical shear stress suggests that it could be a key determinant also in the development of cardiovascular diseases. As previously mentioned, endothelial cells with reduced PALMD expression failed to form the perinuclear actin cap upon flow-induced shear stress, resulting in nuclear shape defects and nuclear misalignment from the flow direction 54. Since the single nucleotide polymorphism rs7543130 in PALMD gene causes reduced PALMD expression in patients with calcific aortic valve stenosis (CAVS), the loss of perinuclear actin cap could participate to the still unknown PALMD-mediated mechanism responsible for the development of CAVS 54.

Finally, an emerging area of investigation is the role of actin cap alterations in cancer progression and metastasis, where these changes could play a primary role.

As mentioned, the actin cap is usually disorganized or completely absent in cancer cells of various origins, which is consistent with its role in proper differentiation.

Theoretically, the disruption of the actin cap could boost transformed phenotypes in multiple ways:

i. By resulting in rounded and enlarged nuclei with reduced stiffness, associated with increased aggressiveness and metastatic potential thanks to an increased nuclear deformability 117-119 as well as immune evasion 120-122.

ii. By inducing a mesenchymal-to-amoeboid transition, in turn facilitating migration in complex environments.

iii. By reducing the dependency of cells on growth in stiff and rigid environments, ultimately allowing survival in soft and liquid matrices - condition typically encountered during dissemination from the primary tumor to metastatic sites.

Of note, cancer cells forced to actin cap restoration through MET silencing/YAP activation, not only switched from fast amoeboid migration to slow and polarized motility, but also acquired matrix rigidity dependency, failing to grow embedded in matrigel or cultured in low-attachment conditions 15. In line, MET overexpression has been found to correlate with altered nuclear size, nuclear irregularities and nucleolar prominence in renal carcinoma patients 123,124. All these phenotypes indicate the clinical relevance of an actin cap alteration, with implications in terms of cancer progression and metastasis.

Interestingly, normal stromal cells are stiffer than cancer cells both in adherent conditions, where this property correlates with the presence of the actin cap, and upon detachment from the substrate, despite the loss of the actin cap. Moreover, normal cells are significantly softened by the addition of stress fibers inhibitors. These data may suggest that traces of the actin cap presence persist in the suspended state and biomechanical analysis may provide indirect evidence about the actin cytoskeleton of suspended cells, allowing to discriminate between a cancer versus normal background 125.

The mechanisms driving actin cap deregulation in cancer remain poorly defined and are likely highly context-dependent. Strikingly, despite sustained oncogenic nuclear YAP activity in many tumor cells 126, the actin cap is consistently disrupted or lost. This indicates that YAP activation is not sufficient to preserve actin cap integrity and suggests that tumor-specific regulatory programs may actively uncouple YAP signaling from cytoskeletal organization. Such uncoupling highlights a critical layer of context-dependent control over nuclear architecture in cancer.

The formation of the actin cap could also correlate with cell response to anti-cancer therapies and drug resistance.

For example, microtubule targeting drugs induce a specific biomechanical response in prostate cancer cells, primarily correlated with the intensification or reduction of perinuclear actin cap fibers, likely through a cross-talk between microtubule and actin cytoskeleton network. Indeed, while vinflunine (VFL), an alkaloid that inhibits the assembly of microtubules, enhanced actin caps formation, leading to cell stiffening occurring especially in the nuclear region, the microtubule stabilizer docetaxel (DTX) produced an opposite effect, characterized by cell softening and reduction of perinuclear actin fibers 127. Nuclear stiffening has been observed also in polyploid giant cancer cells (PGCCs) derived from MDA-MB-231 triple negative breast cancer cells, that fail in nuclear division. PGCCs are often observed in tumors *in vivo* and have been linked to chemoresistance and stemness-like features. In MDA-MB-231 treated with paclitaxel (PTX), the number of PGCCs significantly increased along with the organization of the actin cap, responsible for a directional migration and stiff nuclei 128. More generally, it would be interesting to study the involvement of the actin cap in the acquisition of drug tolerance, which anticipates the development of chemoresistance driving tumor relapse. Indeed, drug-tolerant persister cells (DTPs) are characterized by increased FAK/YAP signaling and enhanced stress fibers, suggesting a role for the actin cap in this context 129.

Finally, emerging evidence highlights the role of cancer-cell stiffening in the response to immunotherapy. Indeed, the remarkable softness of cancer cells, resulting from the poor organization of apical stress fibers, has been identified as a strategy for immune evasion, as it hinders proper pore formation in the plasma membrane during perforin-dependent cytotoxicity 120. In line, key mediators involved in classic EMT such as myocardin related transcription factors (MRTFs) were found to sensitize tumor cells to the immune response by thickening actin fibers. Intriguingly, very recent work by Wang et al. described in metastatic lung adenocarcinoma an atypical EMT program driven by TGF-β, in which cells initially undergo a classic EMT but then transition into a cortical actin-rich, stress fiber-poor rounded morphology driven by gelsolin expression 130. Although the morphological profile is fully reminiscent of classical amoeboid cells, it was found to be associated with growth arrest (dormancy). Most importantly, this phenotypical shift, by lowering cell stiffness, allowed cells to escape immune surveillance during metastasis 130.

Altogether, these findings highlight again the idea of an interplay between actin stress fibers, EMT and mesenchymal-to-amoeboid transition in the metastatic microenvironment that is more complex than previously thought 122. In this perspective, therapeutic strategies that counteracts cell softening, such as cholesterol depletion, have been already proved to enhance T-cell immunotherapy 121, suggesting that drugs acting on perinuclear actin fibers could represent a powerful tool to modulate mechanical immune checkpoint in cancer cells.

To sum up, the detection of actin cap aberrations in the clinical setting might be more relevant than previously thought. On one side, perinuclear actin cap loss could represent a major driver of progression due to the relation with abnormal nuclear phenotypes, calling for the implementation of targeted therapies stimulating perinuclear actin cap restoration. On the other, the detection of actin cap alterations in patient-derived cells could represent a biomarker of certain diseases and/or indication of poor prognosis. In this perspective, it becomes indispensable to discriminate between pathologies where actin cap aberrations are a side consequence of cell structural defects that prevents actin cap alignment (e.g. laminopathies), and conditions in which the diseased phenotype revolves around actin cap loss and its repairing displays a therapeutic effect.

## Figures and Tables

**Table 1:** Perinuclear actin cap structural proteins and their functions.

Actin cap structural protein	Function	Phenotype upon inhibition	References
Nesprin-1/2	Contacts actin filaments and SUN1/2 proteins in the LINC complex	Abrogation of the actin cap, nuclear expansion (MEFs, MCF10A)	(15,17)
Nesprin-3	Indirectly contacts actin filaments via interaction with nesprin1/2 and plectin ABDs	Impairment of shear-induced actin cap formation (C2C12)	(8)
SUN1/2	Contacts nesprins and nuclear lamina in the LINC complex	Actin cap lightening (VSMCs), abrogation of the actin cap (MCF10A)	(15,18)
Lamin A/C	Nuclear lamina component	Abrogation of the actin cap, increase in nuclear volume and thickness (MEFs)	(6,8,17)
α-actinin	Cross-links actin cap fibers	Reduction of actin cap fibers thickness and organization, change in shape and nuclear positioning (MEFs)	(9)
Myosin II	Provides contractility to actin cap fibers	Disorganization or diminishment of actin cap fibers; abrogation of nuclear shape regulation (MEFs)	(6,9)
Refilin B	Converts filamin A from an F-actin branching to an F-actin bundling protein to organize the actin cap	Disrupted actin cap organization, increased nuclear height (NIH3T3)	(19)
Filamin A	Organizes the actin cap when bound to refilins	Disrupted actin cap organization, increased nuclear height (NIH3T3)	(19)
Vinculin, zyxin, talin	Focal adhesions components	Loss of shear-stress-induced actin cap formation (MEFs)	(8)

**Table 2:** List of diseases potentially involving the perinuclear actin cap and their associated putative mechanisms.

Disease spectrum	Disease		Observation/putative mechanism	References
Genetic disorders	Laminopathies	Emery-Dreifuss muscular dystrophy (EDMD)	Actin cap loss due to depletion of lamin A/C or concomitant LINC complex genes mutations contribute to nuclear deformation in laminopathic fibroblasts	(6,7,17,29,108,112)
		Hutchinson-Gilford progeria syndrome (HPGS)		
	Huntington disease (HD)		Actin cap loss in skin fibroblasts of HD patients is linked with nuclear alteration and likely involved in multisystem effects of HD in nonneuronal tissues	(109)
	Spondylocarpotarsal synostosis syndrome (SCT)		Case report in SCT patient with homozygous frameshift mutation in *RFLNA* gene. Putative role of the actin cap in proper development and growth of the vertebral column	(110)
Developmental defects	MSCs aberrant differentiation		Alterations of actin cap in MSCs boost cell proliferation likely impacting on their differentiation process	(38)
	ESCs aberrant differentiation		A proper differentiation of hESCs requires the formation of an actin cap to achieve correct nuclear shaping	(115)
	Myoblasts aberrant differentiation		HP1-gamma inhibition may impact on actin cap formation resulting in altered myotubes differentiation	(116)
Cardiovascular diseases	Calcific aortic valve stenosis (CAVS)		PALMD-dependent alterations of the actin cap impair nuclear resilience in endothelial cells in response to mechanical stress as well as nuclear alignment in mouse aorta and patient samples	(54)
Cancer	Gastrointestinal cancer		Hyperactivation of MET in cancer cells induces YAP inhibition leading to actin cap loss, resulting in the acquisition of aggressive amoeboid-like behavior and capability to grow in lack of adhesion	(15)
	Breast cancer		Polyploid giant cancer cells (PGCCs) display actin cap restoration as putative mechanism of chemoresistance	(128)
	Prostate cancer		Actin cap status varies along with cell stiffness upon administration of different anti-cancer drugs acting on microtubules, likely impacting on the global response to treatment	(127)
Ageing	Physiological ageing		Actin cap loss induced by YAP depletion upon natural decline impacts on nuclear integrity, cGAS activation and senescence	(31)
